# Supplementary material for: Flexi-YOLO: A lightweight method for road crack detection in complex environments
Source: PLoS One. 2025 Jun 16;20(6):e0325993. doi: 10.1371/journal.pone.0325993 (PMC12169580; doi:10.1371/journal.pone.0325993)
Supplement: Attachment — Submitted filename: Response to Reviewers.docx [file pone.0325993.s001.docx]

Response to Reviewers

**Title:** **Flexi-YOLO: A Lightweight Method for Road Crack Detection in Complex Environments**

**Manuscript Number: PONE-D-24-58741**

**Author:** **Jiexiang Yang ^*,+^, Renjie Tian ^+^, Zexing Zhou , Xingyue Tan and Pingyang He**

First of all, the authors would like to express their sincere thanks to the reviewers and editors for helpful comments and suggestions. The explanation of the modifications as well as corrections in this revision can be arranged as follows.

The comments of the reviewers are listed in [comment] in italics below. Our reply is in normal font in [reply]. The modified content is given in [modified] in normal font. The modification details are tracked in detail with the latexdiff given on the official website and named Revised Manuscript with Track Changes.pdf . The modifications or additions to the manuscript are marked in blue text.

**Response to Journal Requirements**

**[Comment]***1.Please ensure that your manuscript meets PLOS ONE's style requirements, including those for file naming. The PLOS ONE style templates can be found at* [*https://journals.plos.org/plosone/s/file?id=wjVg/PLOSOne_formatting_sample_main_body.pdf*](https://journals.plos.org/plosone/s/file?id=wjVg/PLOSOne_formatting_sample_main_body.pdf) *and* [*https://journals.plos.org/plosone/s/file?id=ba62/PLOSOne_formatting_sample_title_authors_affiliations.pdf*](https://journals.plos.org/plosone/s/file?id=ba62/PLOSOne_formatting_sample_title_authors_affiliations.pdf)

**[Reply]**

Thank you for your reminding. We have combined the templates in the official website to make the manuscript typesetting to ensure that the manuscript meets the style requirements of PLoS One. Thank you for your reminding of the manuscript format and file naming. If you have any suggestions, please contact us by email. Thank you.

**[Comment]** *2. PLOS requires an ORCID iD for the corresponding author in Editorial Manager on papers submitted after December 6th, 2016. Please ensure that you have an ORCID iD and that it is validated in Editorial Manager. To do this, go to ‘Update my Information’ (in the upper left-hand corner of the main menu), and click on the Fetch/Validate link next to the ORCID field. This will take you to the ORCID site and allow you to create a new iD or authenticate a pre-existing iD in Editorial Manager.* **[Reply]**

Thank you for your reminding. We have verified ORCID in the Edit Manager and achieved this.

**[Comment]** *3. Please note that PLOS ONE has specific guidelines on code sharing for submissions in which author-generated code underpins the findings in the manuscript. In these cases, we expect all author-generated code to be made available without restrictions upon publication of the work. Please review our guidelines at* [*https://journals.plos.org/plosone/s/materials-and-software-sharing#loc-sharing-code*](https://journals.plos.org/plosone/s/materials-and-software-sharing#loc-sharing-code) *and ensure that your code is shared in a way that follows best practice and facilitates reproducibility and reuse.*

**[Reply]**

Thank you for your reminder. We have uploaded the relevant code to GitHub during submission, and the repository is available at: https://github.com/Noceiling1/code.git. We hope that providing the complete code resources will assist the editors and reviewers in better evaluating the research work presented in our manuscript. Should you have any questions during the review process, we are available to provide further clarification via email at any time.

**[Comment]** *4. Thank you for stating the following financial disclosure:
This work was supported by the National Natural Science Foundation of China under Grants (62276034、62306052), Group Building Scientific Innovation Project for Universities in Chongqing (CXQT21021), Joint Training Base Construction Project for Graduate Students in Chongqing (JDLHPYJD2021016), and Science and Technology Research Program of Chongqing Municipal Education Commission (KJQN202100712).*

*Please state what role the funders took in the study.  If the funders had no role, please state: ""The funders had no role in study design, data collection and analysis, decision to publish, or preparation of the manuscript.""
If this statement is not correct you must amend it as needed.
Please include this amended Role of Funder statement in your cover letter; we will change the online submission form on your behalf.*

**[Reply]**

Thank you very much for the rigorous and meticulous inspection and requirements of the journal in terms of financial disclosure. For the sponsor's statement, "the sponsor has not participated in research design, data collection and analysis, decision to publish or write manuscripts, and the sponsor only supports the hardware equipment and computing support of the experiment and the payment of publishing layout fees."

**[Comment]** *5. We note that your Data Availability Statement is currently as follows: All relevant data are within the manuscript and its Supporting Information files.*

*Please confirm at this time whether or not your submission contains all raw data required to replicate the results of your study. Authors must share the “minimal data set” for their submission. PLOS defines the minimal data set to consist of the data required to replicate all study findings reported in the article, as well as related metadata and methods (*[*https://journals.plos.org/plosone/s/data-availability#loc-minimal-data-set-definition*](https://journals.plos.org/plosone/s/data-availability#loc-minimal-data-set-definition)*).*

*For example, authors should submit the following data:*

*- The values behind the means, standard deviations and other measures reported;
- The values used to build graphs;
- The points extracted from images for analysis.*

*Authors do not need to submit their entire data set if only a portion of the data was used in the reported study.*

*If your submission does not contain these data, please either upload them as Supporting Information files or deposit them to a stable, public repository and provide us with the relevant URLs, DOIs, or accession numbers. For a list of recommended repositories, please see* [*https://journals.plos.org/plosone/s/recommended-repositories.*](https://journals.plos.org/plosone/s/recommended-repositories.)

*If there are ethical or legal restrictions on sharing a de-identified data set, please explain them in detail (e.g., data contain potentially sensitive information, data are owned by a third-party organization, etc.) and who has imposed them (e.g., an ethics committee). Please also provide contact information for a data access committee, ethics committee, or other institutional body to which data requests may be sent. If data are owned by a third party, please indicate how others may request data access.*

**[Reply]**

Thank you very much for your reminding about the dataset. The data supporting the findings of this study are openly available in Zenodo at [https://doi.org/10.5281/zenodo.15019275]. For further inquiries, please contact the corresponding author.

**[Comment]** *6. We are unable to open your Supporting Information file Flexi-YOLO.tex. Please kindly revise as necessary and re-upload.*

**[Reply]**

Sorry about the problem with tex files. We have updated the openable integrated tex file package.

**Response to Reviewer #1**

**[Comment]** *Language Correction:*

*Abstract:*

*Rephrase for conciseness and impact. For instance:*

*"Road crack detection is vital for infrastructure maintenance and public safety, yet complex backgrounds and irregular crack shapes challenge real-time, efficient, and accurate detection. This paper proposes Flexi-YOLO, a lightweight and robust model based on YOLOv8, incorporating novel components to address these challenges. Key innovations include the Wise-IoU loss function for enhanced regression accuracy and robustness, the DCNv-C2f module for feature transformation, and the Global Attention Module (GAM) for improved global information perception. Flexi-YOLO achieves significant improvements in accuracy, recall, and mAP metrics, offering a cost-effective solution for automated crack detection."*

**[Reply]**

Thank you very much for your suggestion. We have rephrased the summary to make it more concise and highlight the core impact.

**[Modified]**

Road crack detection is crucial for the maintenance of infrastructure and public safety. However, the challenges posed by complex background environments and irregular crack shapes demand real-time, efficient, and precise detection. This paper proposes a lightweight yet robust Flexi-YOLO model based on the YOLOv8 algorithm. We designed Wise-IoU as the model's loss function to optimize the regression accuracy of its bounding boxes and enhance robustness to low-quality samples. The DCNv-C2f module is constructed for the transformation and fusion of feature information, allowing the convolutional kernels to adapt to the complex shape characteristics of cracks dynamically. A Global Attention Module (GAM) is integrated to improve the model's perception of global information. The AKConv convolution operation is employed to adaptively adjust the size of convolutions, further enhancing local feature capturing. Additionally, a lightweight network design is implemented, establishing G-Head (Ghost-Head) as the detection head to optimize the issue of feature redundancy. Experimental results show that Flexi-YOLO achieves an accuracy increase of 2.7% over YOLOv8n, a recall rate rise of 4.7%, a mAP@0.5 improvement of 5.3%, a mAP@0.5-0.95 increase of 3.9%, a decrease of 0.5 in GFLOPS, and an F1 score improvement from 0.80 to 0.84. Flexi-YOLO offers higher detection accuracy and robustness and meets the industrial demands for lightweight real-time detection and lower application costs, providing an efficient and precise solution for the automated detection of road cracks.

**[Comment]** *Introduction:*

*Rewrite to engage the reader and highlight the importance of the work globally, not just in a single country. Example:*

*"Road networks form the backbone of global infrastructure, critical to economic growth and societal well-being. However, environmental stressors and prolonged usage lead to structural deterioration, with cracks posing risks to safety, durability, and usability. Timely and accurate crack detection is essential to mitigate these issues, reduce maintenance costs, and ensure sustainability. This paper addresses these challenges by proposing an innovative, lightweight model tailored for real-time crack detection in diverse and complex environments."*

**[Reply]**

Thank you very much for your comments. We restated the content of the introduction. According to your suggestions, we reflected the background and importance of our work on a global scale rather than just discussing one country.

**[Modified]**

Roads are a critical component of global infrastructure and are essential for the long-term stable development of the world economy. However, due to prolonged use and various environmental factors, infrastructure structures inevitably experience aging, with concrete structures particularly susceptible to cracking [1]. These cracks affect road durability, safety, and usability, reduce their load-bearing capacity, shorten their lifespan, and even threaten user safety [2].According to the World Health Organization's 2023 report, approximately 1.19 million people die each year due to road traffic accidents, with nearly 3,400 deaths per day and up to 50 million injured. Among these, individuals aged 5 to 29 represent the primary age group for road accident fatalities, which is also a crucial demographic for nations' current and future economic development, bearing the responsibility for significant economic construction.

**[Comment]** *Related Work:*

*Ensure smooth transitions between traditional methods and deep learning advancements.*

**[Reply]**

Thank you for your professional comments on our article. We have optimized the relevant work to make the transition between traditional methods and deep learning methods more smooth and natural.

**[Modified]**

In recent years, with the rapid development of computer vision, researchers have provided new technological directions for crack detection, which can be broadly divided into two categories. The first category is based on traditional digital image processing, primarily relying on manual feature discrimination. This involves designing specific feature recognition criteria to facilitate identification [15-17]. For instance, Kim J et al. (2003)[18] achieved crack detection and localization through structural frequency changes, Nguyen et al. (2014) [19] utilized the geometric properties of cracks in images to detect crack edges, and Jin et al. (2018) [20] proposed a method for pavement crack detection that integrates directional gradient histograms with watershed algorithms. However, traditional image processing methods struggle to meet crack detection's precision and time requirements [21]. The second category is based on deep learning theory, employing convolutional neural networks for autonomous feature learning, thus achieving target detection of cracks. Deep learning has emerged as a mainstream detection method in recent years, characterized by high automation, intelligence, accuracy, and robustness, and has been widely adopted by scholars [22-24]. Utilizing deep learning methods reduces detection errors caused by human subjectivity and addresses the limitations of traditional image processing methods in recognizing complex backgrounds in practical engineering contexts.

**[Comment]***Expand on limitations of existing YOLO-based methods and how Flexi-YOLO surpasses them.*

**[Reply]**

Thank you for your concern about this aspect of our manuscript. We have added more literature review in the introduction based on the limitations and defects of Yolo algorithm, and briefly described the work content of this paper in the key part to highlight the advantages of Flexi Yolo.

**[Modified]**

In the field of crack detection, there are typically two stages: the first involves traditional detection methods. However, with the advancement of artificial intelligence, deep learning detection is gradually replacing traditional methods. While deep learning models have made significant progress in road crack detection, they still face several key challenges. Factors such as background noise, surface texture, lighting shadows, crack edges, and the cracks' shape, size, depth, and moisture often lead to false positives and missed detections, increasing the difficulty of detection [7]. These factors require models to not only adapt to different background noise but also possess the capability to handle disturbances from surface textures and shadows. Road safety work is often conducted using mobile devices, which typically have limited computational power and lower application costs. Therefore, it is essential to ensure the model's lightweight and flexibility to accommodate such devices. Two-stage detection algorithms based on deep learning generally require large datasets and high computational costs, while single-stage detection methods offer faster speeds and smaller sizes. With the development of the YOLO series, the models' inference speed and lightweight nature have been further enhanced. Comparing YOLOv8, YOLOv9, and YOLOv10, it is found that YOLOv8 maintains lower computational costs while ensuring accuracy and inference speed [8]. Although YOLOv8 demonstrates strong multi-scale feature extraction capabilities, its detection accuracy for small objects and defect cracks is relatively low, leading to missed and false detections. The main reason is that as the network depth increases, some shallow feature information becomes difficult to retain [9].

In response to the aforementioned issues, this paper proposes the Flexi-YOLO model, which is based on the YOLOv8 algorithm and tailored explicitly for road crack detection, effectively addressing current challenges in identifying road cracks. This work aims to overcome the difficulties in road crack detection by achieving the following objectives:

Develop a lightweight model suitable for deployment on devices with limited computational resources, design a backbone network that enhances the capability to extract features of complex geometrical shapes of cracks, integrate attention mechanisms and loss functions to improve the model's detection accuracy and robustness, and redesign a detection head to reduce feature redundancy and computational load, ensuring the model's lightweight nature to meet industrial requirements.

The following are the main points of discussion in this article:

1.Introducing variable convolution DCNv3 in the C2f module enhances the feature extraction of irregularly shaped cracks, which effectively solves the problem of difficult recognition caused by the specificity of crack features (length is much larger than width).

2. Replaced part of the convolution operations with variable kernel convolution AKConv, redesigned the initial sampling points, and utilized the adjustable characteristics of variable convolution kernels to adapt dynamically to the complex geometrical shapes of cracks, strengthening the representation of local features and the fusion capabilities of the backbone network's features.

3. Fusing the GAM attention mechanism in the neck network, utilizing its powerful global information extraction capability, effectively retains the image detail information and enhances the relevance of the global information, thus reducing the leakage and misdetection rates of the model.

4. Design a new lightweight detection head G-head, reduce the redundant features in the model, reduce the computational complexity of the model while ensuring the accuracy, so that the model can be adapted to low-computing-power devices.

5. Utilized the Wise-IoU loss function to optimize boundary box regression, enhancing the model’s adjustment to low-quality sample parameters and improving its robustness and accuracy in processing boundary boxes.

**[Comment]** *Methodology:
Clarify technical terms like “Wise-IoU,” “G-Head,” and “AKConv” with succinct explanations. Avoid jargon overload.*

**[Reply]**

We think this is a good comment, and based on your comment, we have briefly elaborated on the “Wise-IoU,” “G-Head,” and “AKConv” in 3.2.1. Adaptive Dynamic Feature Extraction Trunking, and 3.2.3 Lightweight Detection Header in the paper.

specifically:

AKConv adjusts the sampling position of irregular convolutional kernel by novel coordinate generation algorithm and offset mechanism, and dynamically adjusts the number and shape of kernel parameters according to the specific needs, so as to enhance the feature adaptability and improve the responsiveness to the complex geometry and changing targets.

Head: The decoupling head of YOLOv8 is merged into the coupling head, and the ordinary convolution operation is replaced by the lighter Ghostconv operation, which significantly reduces the number of parameters and computation of the detection head.

Wise-IoU: It is a loss function that adaptively adjusts the proportion of positive and negative samples and regression coefficients. By reducing the harmful gradient generated by low-quality samples, the model is able to focus on normal-quality anchor frames, which realizes the balance between high-quality and low-quality data and thus improves the detection performance.

**[Comment]** *More Literature Review:
Discuss advancements in deep learning for infrastructure health monitoring, such as applications beyond road cracks (e.g., bridge, pipeline, and building inspections).*

*Include recent works utilizing YOLO-based architectures in different domains to position Flexi-YOLO in the broader context.*

**[Reply]**

Thank you very much for your valuable suggestions. We have carefully read the relevant literature according to your comments, defined and sorted out the relationship between road cracks through concrete and bridges and tunnels in the relevant work, and used the three excellent literatures you provided for reference. In the relevant work, we also supplemented the progress of pipeline detection, mine crack detection and bridge deck crack detection, and also fully referred to the literature provided by you for learning, making our manuscript more sufficient and convincing.

**[Modified]**

Deep learning-based detection can be broadly categorized into two types: two-stage detection algorithms and single-stage detection algorithms. The former first generates a series of candidate regions and then performs classification and bounding box regression on these candidate regions. It is represented by R-CNN, Fast R-CNN, Faster R-CNN, and Mask R-CNN [25-28]. The latter predicts the categories and bounding boxes directly on the image without the need to generate candidate regions represented by You Only Look Once (YOLO) [29-30]. A. Shiuly et al[31] compared six models for predicting concrete on compressive strength and demonstrated the effectiveness of deep learning models for cement mortar and concrete compressive strength prediction.Cha et al. (2017)[32] used CNN in combination with the sliding window technique to scan the test images to make the model excel at detecting thin cracks under illumination conditions. Hacı efendioğlu et al.(2022)[33] used Faster R-CNN to study crack detection to conclude that lighting conditions have the highest impact on crack detection. Xu et al.(2022)[34] found that the former is better than the latter under certain conditions by comparing the detection of cracks with Faster R-CNN and Mask R-CNN, but the two algorithms require large and complex datasets. To address these problems, researchers have proposed various optimization strategies. For example, Zhang et al.(2020)[35] introduced MobileNetV2 and CBAM into YOLOv3 to reduce the network parameters. Yu et al.(2021)[36] pruned YOLOv4 to significantly increase the detection rate while ensuring the accuracy to contribute to the UAV crack detection. Chen et al.(2022)[37] introduced the Ghost module and CA attention mechanism in YOLOv5 for the backbone network to effectively improve the detection of coal cracks, which contributes to the construction of intelligent mines. Chen et al. (2022)[38] proposed a sample enhancement strategy based on cycle-GAN, which uses a migration learning strategy to incorporate the Transformer attention mechanism in YOLO v5 to help the detection of cracks by drones. Transformer attention mechanism, to help the network find the region of interest in the complex background, to improve the detection and identification of small-scale defects, for the pipeline defects task to solve the overfitting problem brought by small samples and large models. Tran et al. (2023)[39] compared the mainstream models, selected YOLOv7, and proposed a U-Net model that can detect and segment bridge deck cracks in a fast and high-precision way. detection and segmentation of bridge surface cracks. Xiong et al.(2024)[40] incorporated the GAM attention mechanism and Wise-IoU into YOLOv8 to study the bridge surface cracks and found that the GAM attention mechanism has a great effect on crack detection. Wang et al.(2023)[41] optimized YOLOv8 with simSPPF using the spatial pyramid pooling layer in YOLOv8 and introduced the dynamic large convolutional kernel LSK attention mechanism, making the model more effective for pavement defect detection. Xu et al.(2025)[42] designed three plug-and-play modules DGLFG, SCCFF, and ISPP based on YOLOv8, which significantly improved the feature learning ability of aerial photography for small object detection. Liu et al.[43] introduced the C2f_AK module in the neck network to enhance feature fusion, significantly improved the detection head, and reduced parameters to achieve model lightweighting.

**[Comment]***Highlight shortcomings of current methods, like high computational requirements, sensitivity to noise, or lack of adaptability to varying crack shapes.*

**[Reply]**

Thank you for your suggestion. We added the limitations of Yolo and yoov8 in the introduction and at the end of Section 3.1.

**[Modified]**

Introduction: In the field of crack detection, there are typically two stages: the first involves traditional detection methods. However, with the advancement of artificial intelligence, deep learning detection is gradually replacing traditional methods. While deep learning models have made significant progress in road crack detection, they still face several key challenges. Factors such as background noise, surface texture, lighting shadows, crack edges, and the cracks' shape, size, depth, and moisture often lead to false positives and missed detections, increasing the difficulty of detection [7]. These factors require models to not only adapt to different background noise but also possess the capability to handle disturbances from surface textures and shadows. Road safety work is often conducted using mobile devices, which typically have limited computational power and lower application costs. Therefore, it is essential to ensure the model's lightweight and flexibility to accommodate such devices. Two-stage detection algorithms based on deep learning generally require large datasets and high computational costs, while single-stage detection methods offer faster speeds and smaller sizes. With the development of the YOLO series, the models' inference speed and lightweight nature have been further enhanced. Comparing YOLOv8, YOLOv9, and YOLOv10, it is found that YOLOv8 maintains lower computational costs while ensuring accuracy and inference speed [8]. Although YOLOv8 demonstrates strong multi-scale feature extraction capabilities, its detection accuracy for small objects and defect cracks is relatively low, leading to missed and false detections. The main reason is that as the network depth increases, some shallow feature information becomes difficult to retain [9].

The end of Section 3.1: Despite the exceptional performance of YOLOv8, effectively handling occluded objects and cluttered scenes and adapting to overlapping or various crack shapes remains a long-term challenge for the YOLO family [44].

**[Comment]** *Incorporate examples of lightweight models applied in mobile and edge computing for infrastructure maintenance, emphasizing how Flexi-YOLO aligns with these trends.*

**[Reply]**

Thank you for noticing this detail and providing valuable feedback. We have supplemented our literature review with more relevant studies that improve fundamental algorithms and creatively integrate basic modules to endow our model with such characteristics, which aligns perfectly with our research trends. Inspired by the work on detection head optimization[Liu, Chuanyao, et al. "YOLO-Powerlite: a lightweight YOLO model for transmission line abnormal target detection." IEEE Access (2024).], we investigated the parameter count of the YOLOv8 model. Experimental results indicate that the decoupled head design accounts for a significant portion of the model's parameters. This presents a substantial optimization opportunity; therefore, we are focusing our research on redesigning the detection head to achieve model lightweighting. The new detection head significantly reduces the information redundancy caused by the attention mechanism's extensive collection of global information and the original detection head's parameter count, enabling the model to be lightweight and adaptable to low computing power.

**[Modified]**

Wang et al.(2023)[41] optimized YOLOv8 with simSPPF using the spatial pyramid pooling layer in YOLOv8 and introduced the dynamic large convolutional kernel LSK attention mechanism, making the model more effective for pavement defect detection. Xu et al.(2025)[42] designed three plug-and-play modules DGLFG, SCCFF, and ISPP based on YOLOv8, which significantly improved the feature learning ability of aerial photography for small object detection. Liu et al.[43] introduced the C2f_AK module in the neck network to enhance feature fusion, significantly improved the detection head, and reduced parameters to achieve model lightweighting.

**[Comment]***Literature references suggested for manuscript optimization, Paper1: “Study on the use of different machine learning techniques for prediction of concrete properties from their mixture proportions with their deterministic and robust optimisation,” Paper 2: ” Revolutionizing concrete analysis: An in-depth survey of AI-powered insights with image-centric approaches on comprehensive quality control, advanced crack detection and concrete property exploration,” and Paper 3: ” Assessing compressive strengths of mortar and concrete from digital images by machine learning techniques,”.*

**[Reply]**

We sincerely thank you for your valuable comments. We carefully read the literature, and added references and citations about the three literatures in the introduction and related work of the revised draft. A richer literature review makes our article more complete.

**[Modified]**

Introduction: If road cracks are not maintained and repaired, minor cracks can evolve into deep fissures and potholes, leading to greater economic losses. As one of the primary materials for transportation infrastructure such as roads, bridges, and tunnels, the health status of concrete structures is a primary concern for infrastructure. Enhancing the strength and durability of concrete to improve the safety of foundational structures is a reliable method [3], while crack detection serves as a key indicator for assessing the condition of concrete structures and monitoring the safety and maintainability of infrastructure. Utilizing deep learning for detection is another mainstream approach [4], playing a vital role in subsequent road maintenance and repairs. Therefore, timely detection and assessment of road cracks are necessary to reduce future repair and maintenance costs [5].

In Section 2.2:Deep learning-based detection can be broadly categorized into two types: two-stage detection algorithms and single-stage detection algorithms. The former first generates a series of candidate regions and then performs classification and bounding box regression on these candidate regions. It is represented by R-CNN, Fast R-CNN, Faster R-CNN, and Mask R-CNN [25-28]. The latter predicts the categories and bounding boxes directly on the image without the need to generate candidate regions represented by You Only Look Once (YOLO) [29-30]. A. Shiuly et al[31] compared six models for predicting concrete on compressive strength and demonstrated the effectiveness of deep learning models for cement mortar and concrete compressive strength prediction.Cha et al. (2017)[32] used CNN in combination with the sliding window technique to scan the test images to make the model excel at detecting thin cracks under illumination conditions. Hacı efendioğlu et al.(2022)[33] used Faster R-CNN to study crack detection to conclude that lighting conditions have the highest impact on crack detection. Xu et al.(2022)[34] found that the former is better than the latter under certain conditions by comparing the detection of cracks with Faster R-CNN and Mask R-CNN, but the two algorithms require large and complex datasets. To address these problems, researchers have proposed various optimization strategies. For example, Zhang et al.(2020)[35] introduced MobileNetV2 and CBAM into YOLOv3 to reduce the network parameters. Yu et al.(2021)[36] pruned YOLOv4 to significantly increase the detection rate while ensuring the accuracy to contribute to the UAV crack detection. Chen et al.(2022)[37] introduced the Ghost module and CA attention mechanism in YOLOv5 for the backbone network to effectively improve the detection of coal cracks, which contributes to the construction of intelligent mines. Chen et al. (2022)[38] proposed a sample enhancement strategy based on cycle-GAN, which uses a migration learning strategy to incorporate the Transformer attention mechanism in YOLO v5 to help the detection of cracks by drones. Transformer attention mechanism, to help the network find the region of interest in the complex background, to improve the detection and identification of small-scale defects, for the pipeline defects task to solve the overfitting problem brought by small samples and large models. Tran et al. (2023)[39] compared the mainstream models, selected YOLOv7, and proposed a U-Net model that can detect and segment bridge deck cracks in a fast and high-precision way. detection and segmentation of bridge surface cracks. Xiong et al.(2024)[40] incorporated the GAM attention mechanism and Wise-IoU into YOLOv8 to study the bridge surface cracks and found that the GAM attention mechanism has a great effect on crack detection. Wang et al.(2023)[41] optimized YOLOv8 with simSPPF using the spatial pyramid pooling layer in YOLOv8 and introduced the dynamic large convolutional kernel LSK attention mechanism, making the model more effective for pavement defect detection. Xu et al.(2025)[42] designed three plug-and-play modules DGLFG, SCCFF, and ISPP based on YOLOv8, which significantly improved the feature learning ability of aerial photography for small object detection. Liu et al.[43] introduced the C2f_AK module in the neck network to enhance feature fusion, significantly improved the detection head, and reduced parameters to achieve model lightweighting.

**[Comment]** *Highlighting the Novelty of the Work:
Clearly articulate the novel aspects of Flexi-YOLO in the introduction and conclusion. For example:
*"Flexi-YOLO introduces several novel contributions to road crack detection:
The integration of deformable convolution (DCNv3) in the C2f module enhances feature extraction for cracks with irregular shapes.
Variable kernel convolution (AKConv) dynamically adjusts to complex geometries, strengthening local feature representation.
A lightweight detection head (G-Head) reduces computational costs while preserving accuracy, making the model suitable for deployment on mobile devices.
Wise-IoU optimizes bounding box regression, improving robustness against low-quality samples.
These advancements collectively enhance detection accuracy, speed, and adaptability in diverse environments."*.*

**[Reply]**

Thank you very much for your inspection and suggestions. Our introduction should start from the improvement of the model and highlight the novelty of Flexi Yolo, which is particularly important. Therefore, we revised the text and clearly elaborated the innovation of Flexi Yolo, and changed its position to the introduction part, so as to make our manuscript more characteristic, highlight the academic characteristics and the integrity of the paper structure according to your suggestions.

**[Modified]**

The following are the main points of discussion in this article:

1.Introducing variable convolution DCNv3 in the C2f module enhances the feature extraction of irregularly shaped cracks, which effectively solves the problem of difficult recognition caused by the specificity of crack features (length is much larger than width).

2. Replaced part of the convolution operations with variable kernel convolution AKConv, redesigned the initial sampling points, and utilized the adjustable characteristics of variable convolution kernels to adapt dynamically to the complex geometrical shapes of cracks, strengthening the representation of local features and the fusion capabilities of the backbone network's features.

3. Fusing the GAM attention mechanism in the neck network, utilizing its powerful global information extraction capability, effectively retains the image detail information and enhances the relevance of the global information, thus reducing the leakage and misdetection rates of the model.

4. Design a new lightweight detection head G-head, reduce the redundant features in the model, reduce the computational complexity of the model while ensuring the accuracy, so that the model can be adapted to low-computing-power devices.

5. Utilized the Wise-IoU loss function to optimize boundary box regression, enhancing the model’s adjustment to low-quality sample parameters and improving its robustness and accuracy in processing boundary boxes.

**[Comment]** *Presenting the Objectives:
The objectives should be clearly stated at the end of the introduction. Example:
*"This work aims to address the challenges of road crack detection by achieving the following objectives:
Develop a lightweight and efficient model suitable for deployment on devices with limited computational power.
Enhance feature extraction for complex crack geometries using deformable convolution and variable kernel techniques.
Improve detection robustness and accuracy with a novel loss function and attention mechanisms.
Reduce feature redundancy and computational load through a redesigned detection head, ensuring real-time performance.
Validate the model's performance in diverse, real-world scenarios to meet industrial demands."**

**[Reply]**

Thank you very much for your careful review of our manuscript and your suggestions about the goal at the end of the introduction. This will make our article clearly inform the readers of the core content and goal of the research in the introduction, and greatly enhance the readability of the article.

**[Modified]**

In response to the aforementioned issues, this paper proposes the Flexi-YOLO model, which is based on the YOLOv8 algorithm and tailored explicitly for road crack detection, effectively addressing current challenges in identifying road cracks. This work aims to overcome the difficulties in road crack detection by achieving the following objectives:

Develop a lightweight model suitable for deployment on devices with limited computational resources, design a backbone network that enhances the capability to extract features of complex geometrical shapes of cracks, integrate attention mechanisms and loss functions to improve the model's detection accuracy and robustness, and redesign a detection head to reduce feature redundancy and computational load, ensuring the model's lightweight nature to meet industrial requirements.

**[Comment]** *Language Refinement:
Avoid repetitive phrases like "complex backgrounds" and "irregular crack shapes." Use synonyms or restructure sentences for variety.
Use active voice for clarity and engagement. For example:
"Flexi-YOLO addresses the limitations of previous methods by leveraging advanced convolutional techniques and attention mechanisms."*

**[Reply]**

Thank you very much for your suggestion. We carefully reviewed the article, reorganized and replaced the overly repetitive language to make the article more readable. We also carefully reviewed the language standardization, and made improvements under the strict requirements of standardization. In addition, where the novelty and conclusion of the research are elaborated, the active voice is used more to highlight the clarity and participation of the writing expression, and the language is optimized to make the article more in line with the writing habits of the English mother tongue. We are very grateful to the reviewers for their accurate detection and suggestions on the details, which makes our article more perfect.

**[Comment]** *Conclusion:
Reiterate the novelty and potential applications of Flexi-YOLO, emphasizing its real-world impact. Example:
"Flexi-YOLO represents a significant step forward in automated road crack detection. Its lightweight design, coupled with state-of-the-art feature extraction and attention mechanisms, ensures high accuracy and adaptability in diverse environments. This model not only meets the demands of real-time applications but also offers a cost-effective solution for infrastructure maintenance, contributing to public safety and sustainability."*

**[Reply]**

Thank you for your valuable comments. For the guidance of the conclusion part, we have optimized the language from brevity and certainty, highlighted the novelty and potential application of Flexi Yolo, and provided an effective method for the application of equipment with limited computing resources, providing technical progress for the research in the field of road diseases.

**[Modified]**

This article presents a novel Flexi-YOLO model applied to road crack detection. The model employs DCNv-C2f adaptive convolution sampling positions and AKConv adaptive convolution kernel sizes within its backbone network to enhance its ability to extract crack features, allowing for more accurate and flexible crack identification. By adding a GAM attention mechanism at the neck of the network, the model can comprehensively capture global information and further fuse detailed features with low-level network characteristics to obtain more feature information. The newly designed detection head mitigates the issue of information redundancy caused by the attention mechanism's extensive global information collection and the increased model size due to the high parameter count and computation in the original detection head, making it challenging to deploy on devices with limited computational resources. The Wise-IoU method assigns different weights based on sample quality, effectively reducing the negative impact of low-quality samples on the model. With these improvements, the YOLOv8 model performs better in handling complex information across various road conditions, demonstrating stability against fine details and interference information. The developed model shows outstanding performance across various metrics, specifically a 2.7% increase in accuracy, a 4.7% increase in recall, a 5.3% improvement in mAP@0.5, a 3.9 improvement in mAP@0.5-0.95, a 0.5 decrease in GFLOPS, and an increase in F1 score from 0.80 to 0.84. This data underscores the superiority of Flexi-YOLO in multiple dimensions. Furthermore, practical detection experiments indicate that our model excels in numerical evaluation and real-world road condition detection. This validates the practicality of our model improvements and provides valuable experience and technological advancement for real-world road crack operations, offering reliable solutions for road aging, safety, and maintenance. Its lightweight design also presents an economically efficient solution for infrastructure maintenance, contributing to public safety and sustainable development.

**Response to Reviewer 2**

**[comment]***The paper presents Flexi-YOLO, an enhanced road crack detection model based on YOLOv8, which improves detection accuracy while maintaining a lightweight design. The manuscript is generally well-structured and contributes valuable insights to the field of road crack detection. However, there are several important issues that need to be addressed before the paper can be accepted and published:
1.The dataset used in the study contains 4,040 images with predefined augmentation techniques. This may limit the model's ability to generalize to diverse road conditions and surface types in real-world applications. A broader, more varied dataset could improve the model’s adaptability across different environments.*

**[Reply]**

We appreciate the valuable suggestions from the reviewers. Over-reliance on predefined augmentation techniques may limit the model's generalization capacity under various road conditions and surface types. In our initial experiments, we indeed did not fully consider this issue, and we are grateful to the reviewers for highlighting it.

To address this problem and enhance the model's performance and generalization capability in practical applications, we have implemented the following measures:

1. Expanded Dataset: To tackle the issue of insufficient diversity in our dataset, we added new data and conducted experiments comparing generalization across multiple datasets. Specifically, we selected crack datasets with different characteristics, including DATA6000, MotorBike, and Drone, enabling the model to train and validate across a wider range of road environments and crack types.

2. Dataset Compatibility and Performance Assurance: We were particularly mindful that merging incompatible data distributions could lead to decreased model performance. Therefore, we employed stratified sampling and meticulous preprocessing in our multi-dataset experiments to ensure compatibility between datasets. This approach helped avoid potential performance fluctuations due to inconsistent data distributions and ensured the stability and reliability of our experimental results.

3. Model Performance Validation: Through rigorous validation across multiple datasets, we confirmed the robustness of the improved model in different environments. Our enhanced model achieved significant performance improvements across all participating experimental datasets.

4. Enhanced Overfitting Resistance: Further analysis indicated that the proposed augmentation features effectively mitigated overfitting during the model training process, ensuring that the model could handle diverse crack types without relying on specific data characteristics. This was clearly demonstrated in our experimental validations across different datasets, further enhancing the model's robustness.

Through these experiments and optimizations, we established that the improved model not only performs well on specific datasets but also adapts to various practical application scenarios. Therefore, our improvements can be viewed as a genuine enhancement of the model's robustness and generalization capacity within the field of crack detection. This outcome aligns closely with the reviewers' concerns regarding real-world applicability, and we firmly believe that this strategy will make our model more practical and valuable for broader use.

**[Modified]**

In order to comprehensively assess the generalization performance of the Flexi-YOLO model across different datasets, we designed a multidimensional comparative experimental framework. The data sources for the experiment are divided into two parts: 1. The self-constructed Data6000 dataset, consisting of 6,000 images that cover various road scenes (such as urban roads, highways, and rural roads), different lighting conditions (sunny, cloudy, and nighttime), and multiple types of cracks (transverse cracks, longitudinal cracks, and network cracks), sourced from the integrated datasets of the Roboflow website. 2. The RDD2022 dataset [63], which includes four categories of typical road damage: longitudinal cracks (D00), transverse cracks (D10), network cracks (D20), and potholes (D40).

In the experimental design and dataset processing, we adopted a dual-control strategy: Horizontal control involves introducing the RDD2022 dataset to partition it into subsets from different countries and regions, validating the model's adaptability in various areas and environments. Vertical control divides the dataset according to the specific characteristics of road detection scenarios, distinguishing between ground view and aerial view. This further subdivides the RDD2022 dataset into two categories: low-angle motorcycle views (MotorBike) and drone aerial views to address differences in equipment perspectives. Flexi-YOLO model was specially optimized for the geometric features of linear cracks, its parameter update process was not explicitly tailored to network cracks (D20) and potholes (D40). However, the excellent generalization ability of Flexi-YOLO ensures that optimizing D00 and D10 categories does not negatively impact other damage types. Consequently, the other damage types have been excluded from the comparative experiments in this study. Ultimately, three sub-datasets were formed to conduct comparative experiments in different scenarios.

For data partitioning, we divided all datasets into a training set, validation set, and test set in an 8:1:1 ratio to ensure that the model could adequately learn the features during training while preventing the risk of overfitting.

We selected YOLOv8n as the baseline model and conducted a comprehensive evaluation based on four core metrics: Precision, Recall, mean Average Precision at IoU=0.5 (mAP@0.5), and mean Average Precision at IoU from 0.5 to 0.95 (mAP@0.5-0.95).

| Model | Label | Precision | Recall | mAP@0.5 | mAP@0.5-0.95 |
| --- | --- | --- | --- | --- | --- |
| Data6000-v8n | Crack | 0.76 | 0.545 | 0.633 | 0.409 |
| Data6000-Flexi | Crack | 0.76 | 0.605 | 0.669 | 0.455 |
| Motor-v8n | D00 | 0.815 | 0.815 | 0.853 | 0.52 |
|  | D10 | 0.809 | 0.764 | 0.845 | 0.459 |
| Motor-Flexi | D00 | 0.86 | 0.835 | 0.889 | 0.564 |
|  | D10 | 0.865 | 0.796 | 0.873 | 0.504 |
| drone-v8n | D00 | 0.738 | 0.647 | 0.722 | 0.356 |
|  | D10 | 0.799 | 0.684 | 0.733 | 0.38 |
| drone-Flexi | D00 | 0.728 | 0.737 | 0.742 | 0.378 |
|  | D10 | 0.761 | 0.731 | 0.742 | 0.379 |

The experimental results are shown in Table 5, where the Flexi-YOLO model demonstrates significant advantages across three datasets. On the data6000 dataset, Recall improved by 6%, and mAP50_95 increased by 4.6%. In the MotorBrike subset, all detection accuracy metrics improved: for classes D00/D10, Precision (P) increased by 4.5%/5.6%, Recall (R) enhanced by 2%/3.2%, mAP@0.5 rose by 3.6%/2.8%, and mAP@0.5-0.95 grew by 4.4%/4.5%. In the drone dataset, Recall for classes D00/D10 improved by 9%/4.7%, mAP@0.5 increased by 2%/1.9%, and mAP@0.5-0.95 saw enhancements of 2.2%/0.1%. These experimental results strongly confirm that Flexi-YOLO significantly enhances the generalization capability for multi-scenario road damage detection through architectural improvements and optimized training strategies, providing a reliable technical foundation and approach for future engineering deployment.

**[comment]***2.While the model demonstrates strong performance in controlled experiments, there is a lack of real-world validation to assess its robustness under different environmental conditions (e.g., varying lighting, weather, and road types). It is crucial to test the model in non-controlled, field-based scenarios to ensure its practical applicability.*

**[Reply]**

Thank you for the important questions raised by the reviewers regarding the practical applicability of the model. Although our model has performed exceptionally well in controlled experiments, its robustness and suitability in complex, uncontrolled real-world environments still require further validation. To assess the model's robustness in practical settings, we conducted field tests under various weather conditions, lighting scenarios, and road types, including deploying the model on an RTX 1650 graphics card with limited AI computing power. We collected photographs taken on actual roads, capturing images in sunny, cloudy, and rainy conditions, as well as across different types of roads. The experimental results indicate that the model performs well in these real-world scenarios, maintaining stable crack detection despite varying lighting and weather conditions. Our experiments demonstrate that the Flexi-YOLO model possesses strong practicality, enabling it to adapt to crack detection tasks in diverse environmental conditions.

**[comment]**Despite the focus on a lightweight model, the experimental setup relies on high-performance hardware such as the NVIDIA RTX 4090 GPU. This contradicts the paper’s claim of low computational cost, especially for deployment in mobile or resource-constrained environments. It would be helpful to see performance metrics on more typical, low-cost hardware configurations.

**[Reply]**

Thank you for your patient and careful review. We agree with your suggestions on the experimental hardware configuration very much. Some misunderstandings have occurred due to the imprecision of our language. Here we apologize to you. We have conducted a supplementary experiment based on your feedback. In the original text, the "low cost" we mentioned not only refers to the hardware procurement cost, but also includes the resource consumption of the model in the reasoning process In the process of model training, it usually needs a lot of computing resources to optimize the parameters of the model, especially for the training of large-scale data sets, which requires strong computing power. The main purpose of training with RTX 4090 is to accelerate the process. However, the actual detection is usually carried out in the reasoning stage, and the reasoning process does not require expensive computational resources as the training process. Low cost does not only refer to the cost of hardware itself, but also refers to the consumption of computing resources in the reasoning stage. M and gflops can largely reflect the consumption and dependence on hardware resources The smaller the parameter quantity (m), the smaller the occupation of the display memory or memory, and the smaller the calculation quantity gflops, the smaller the consumption of hardware resources That is to say, the smaller the m of the model and the smaller the gflops, the smaller the requirement for hardware cost. Therefore, as long as the M and gflops of the model are small enough, it can represent the low cost of computing resources. Even if a high-performance graphics card is used in training, as long as the reasoning calculation of the model does not depend on a large number of resources, it can be called low cost. Our Flexi Yolo model uses a smaller m and a lower gflops to achieve a higher detection effect than the popular model, and through optimization, the model can run stably on low-power equipment, which proves that the model is low-cost and advanced. Although NVIDIA RTX 4090 GPU is used for experimental training in this study, it does not mean that the model actually needs to consume so many resources. Therefore, we conducted reasoning verification on the model on NVIDIA GTX 1650 low-end graphics card to simulate various devices with similar limited resources. As a common low-end graphics card, GTX 1650 has only 2.9tflops of computing power in theory, which can represent most devices with limited computing power resources. The experimental results show that although the hardware performance is low, the model can still maintain good performance in reasoning effect and accuracy.

**[Modified]**

To validate the feasibility of the Flexi-YOLO model proposed in this paper for practical applications, we deployed and tested the model on an NVIDIA GeForce RTX 1650 graphics card. The RTX 1650 is a cost-effective entry-level GPU with 4GB of memory and 896 CUDA cores, providing a computational power of approximately 2.9 TFLOPS. Its limited performance is suitable for simulating devices with similarly constrained computing capabilities and evaluating our model's performance. To assess the model's robustness, stability, and lightweight performance in various real-world environments, we captured images of road cracks in different settings as test data. In the images, 'a' represents the original image, 'b' corresponds to YOLOv8n, and 'c' denotes Flexi-YOLO.

Fig: Actual Inspection Image

The experimental results presented in Figure indicate that the model demonstrates exceptional inference capabilities on the RTX 1650 graphics card. In practical detection scenarios, the model exhibits strong robustness and detection accuracy. The baseline model, however, is prone to more false positives and missed detections in real-world testing, and it struggles with accurately locating cracks and forming detection anchor boxes. In contrast, Flexi-YOLO performs excellently in real-world detection, particularly in complex environments such as bright light, shadows, and mottled backgrounds, where it still accurately identifies the location and shape of road cracks. The model effectively distinguishes multiple cracks in road images into different subsets while maintaining a low probability of false positives and achieving higher detection accuracy than the baseline model.

Validation through real-world detection confirms the effectiveness of the proposed improvements regarding lightweight design and practical application. The results demonstrate that the model can operate efficiently on resource-constrained devices through reasonable model design and lightweight optimization while maintaining high detection accuracy and real-time performance. This highlights the significant potential application of the proposed method, which meets the requirements for light weight and efficiency in practical engineering contexts.

**[comment]**4.The paper compares the Flexi-YOLO model with YOLOv5, YOLOv7, and YOLOv8, but it does not include other state-of-the-art lightweight models (e.g., transformer-based models or more recent, efficient architectures). Including a broader range of models for comparison would provide a more comprehensive evaluation of the Flexi-YOLO model’s performance and efficiency.

**[Reply]**

Thank you for your professional and pertinent comments on our paper. Due to our negligence, we did not compare the state-of-the-art models of other mainstream architectures. I have benefited from your professionalism and rigor, which has greatly improved the quality of our paper. In response to your suggestion to compare the Flexi-YOLO model with more modern target detection models, we have extended the experimental section to include a comparison with the Transformer-based model RT-DETR.

RT-DETR is specifically designed for real-time target detection tasks and balances accuracy and inference speed. Unlike traditional Transformer-based models (e.g., DETR), which typically face high computational cost and slow inference, RT-DETR introduces an efficient hybrid encoder and a lightweight decoder that significantly reduces latency while maintaining leading detection performance. This makes RT-DETR the preferred choice among the models in the Transformer architecture for real-time processing tasks. We believe that RT-DETR is a suitable comparative model to comprehensively evaluate the advantages of Flexi-YOLO in terms of accuracy, computational efficiency, and inference speed, and for this reason, we have chosen RT-DETR as the For this reason, we choose RT-DETR as a representative model in the Transformer architecture. Through comparative experiments, Flexi-YOLO has obvious advantages in real-time and resource-constrained environments, and is especially suitable for real-world applications with fast response and limited computational resources. Through these comparisons, we are able to clearly demonstrate the advantages of Flexi-YOLO in terms of computational efficiency and real-time detection, especially in application scenarios with lightweight design and low computational cost, showing the superiority over other modern target detection models.

**[Modified]**

To further demonstrate the superiority of the method proposed in this paper, we compared the model with YOLOv5, YOLOv7, YOLOv8, YOLO9, YOLOv10,and RT-DETR as shown in Table .

| Model | Precision | Recall | mAP@0.5 | mAP@0.5-0.95 | GFLOPS | F1 |
| --- | --- | --- | --- | --- | --- | --- |
| YOLOv5n | 0.841 | 0.727 | 0.799 | 0.546 | **4.1** | 0.779 |
| YOLOv7tiny | 0.826 | 0.687 | 0.737 | 0.511 | 13.0 | 0.750 |
| YOLOv8n | 0.88 | 0.735 | 0.808 | 0.614 | 8.1 | 0.808 |
| YOLOv9-t | 0.88 | 0.73 | 0.818 | 0.63 | 10.7 | 0.798 |
| YOLOv10n | 0.842 | 0.728 | 0.806 | 0.608 | 8.2 | 0.780 |
| RT-DETR | **0.915** | 0.736 | 0.831 | 0.653 | 56.9 | 0.81 |
| Ours | 0.907 | **0.782** | **0.861** | **0.653** | 7.6 | **0.84** |

Based on the data in the table, the improved model outperforms others across four or five evaluation metrics: Precision, Recall, mAP@0.5, mAP@0.5-0.95, and F1 score, demonstrating significantly superior detection performance. The experimental results indicate that our proposed model significantly enhances detection capabilities without consuming excessive resources. To visually demonstrate the improved effects of our model, we conducted inference experiments, comparing it with YOLOv5n, YOLOv7tiny, YOLOv8n, YOLOv9t, YOLOv10n, and RT-DETR. We selected a diverse set of cracks with varying scales and shapes as our experimental data, ensuring a large quantity and variety.

Fig: Results of the Inference Experiment

The experimental results are shown in Figure , where a represents YOLOv5n, b represents YOLOv7tiny, c represents YOLOv8n, d represents YOLOv9t, e represents YOLOv10n, f represents RT-DETR, and g represents Flexi-YOLO. The results indicate that the proposed Flexi-YOLO model significantly outperforms other comparative models regarding detection accuracy and localization precision. It is noteworthy that although other models can detect most cracks, they still exhibit various shortcomings in practical applications. For instance, YOLOv5n and YOLOv9t display lower detection accuracy and a high rate of missed detections. Moreover, YOLOv5n, YOLOv8n, YOLOv10n, and RT-DETR demonstrate significant issues with overlapping detection boxes during detection. All comparative models fail to accurately detect targets in complex scenarios such as shadows, low lighting, low contrast, and small cracks, resulting in high missed detection rates. Additionally, these models often redundantly detect inevitable cracks when generating prediction boxes. The high similarity between background noise and crack features leads to frequent false detections. Specifically, the Transformer-based RT-DETR, while slightly superior in detection accuracy compared to other models, suffers from even more severe false and missed detection issues. Its substantial computational load of up to 56.9 GFLOPS places it at a disadvantage in computational efficiency, significantly diminishing its cost-effectiveness.

**[comment]**5.The authors use several image augmentation techniques to improve model robustness. It would be beneficial to briefly discuss how these augmentation methods specifically influence the model’s performance, particularly for detecting cracks under varying conditions (e.g., different weather, lighting, and road types). A deeper analysis of the role and effectiveness of these augmentation strategies would offer valuable insights into how the model generalizes across real-world scenarios. For example, the ideas in these paper can be used as a reference:1.Automatic detection of tunnel lining crack based on mobile image acquisition system and deep learning ensemble model. <https://doi.org/10.1016/j.tust.2024.106124.> 2. Tunnel lining crack detection model based on improved YOLOv5. <https://doi.org/10.1016/j.tust.2024.105713>

**[Reply]**

Thank you very much for your careful review of our manuscript and your valuable comments. The traditional yolov8 backbone network has a poor effect on the feature extraction of the target with highly irregular shape such as cracks. In order to better extract the fracture feature information from the backbone network, the traditional C2F module is improved. By adding an offset to each sampling point through dcnv convolution, the shape and size of each convolution extracted feature can be more similar to the shape and size of the target feature, that is, the dynamic receptive field adaptation, which overcomes the sampling limitation of the traditional convolution fixed geometry. In addition, dcnv's powerful adjustment mechanism is used to improve the focusing ability of the network to the target area, and the nonlinear deformation features, especially various irregular fractures, are captured through offset learning. In this way, C2F layer can generate more representative output each time when extracting features, which helps to improve the performance and expression ability of the network, and is more friendly to the feature extraction of fracture data, which can improve the detection performance of the model. After the dcnv-c2f layer completes the feature expression, akconv performs feature fusion. Akconv dynamically generates convolution kernel weights, and adaptively adjusts convolution parameters according to the context of the input feature map, so as to enhance the expression ability of multi-scale features of the model and suppress background noise. In the backbone network, the details of cracks are preserved as much as possible. Dcnv-c2f and akconv cooperate to enhance the mechanism. The former is responsible for local convolution modeling, and the latter is responsible for multi-scale feature fusion. The two share parameters and complement each other to jointly improve the feature extraction ability of the model for complex cracks

GAM acts on the head of the neck network. After the sppf module at the last layer of the backbone network converts the features of different scales into feature vectors of fixed size, these feature vectors are accepted by gam attention mechanism and re extract the important features to enhance the feature fusion ability of the network. At the same time, the crack information extracted by the backbone network is fully retained and transmitted to the neck network for further processing

**[Modified]**

(1) Feature extraction is a crucial step in model training. We have redesigned the backbone network to enhance the model’s detection performance by extracting crack features at different scales and capturing more details related to crack features. This led to the development of the DCNv-C2f module and the AKConv module, which serve as core components of the backbone network. Traditional convolution typically utilizes fixed-size kernels (e.g., 1×1, 3×3, 5×5) for sampling input feature maps. However, this method often results in suboptimal feature extraction for irregularly shaped targets, exhibiting poor adaptability to unknown variations and limited generalization ability. Fixed-form convolutions have certain limitations when dealing with images that present complex and varied features [50]. Given the diversity of crack shapes and the complexity of backgrounds, traditional convolution operations fail to meet the performance requirements for crack detection. The DCNv-C2f module introduces offsets at each sampling point to achieve dynamically adaptive receptive fields. This mechanism allows each convolution operation to extract features that more closely approximate the actual shape and size of the target features, overcoming the sampling limitations of traditional convolution with fixed geometric shapes. Furthermore, the powerful modulation mechanism of DCNv enhances the network's ability to focus on target regions, enabling the capture of nonlinear deformation features, particularly various irregular cracks. Under this design, the C2f layer generates outputs with greater representational power during each feature extraction, which helps improve the network's performance and expressive capability, making the feature extraction process more attuned to the characteristics of crack data, thereby significantly enhancing the model’s detection performance. As shown in Figure 3, deformable convolution adds a directional parameter to each element of the kernel, allowing the position of sampling points to be dynamically adjusted based on the input content of the image, enabling the kernel to cover a larger area.

(2):To further extract the subtle features of narrow cracks, AKConv is closely integrated with DCNv-C2f. After each DCNv-C2f operation, an AKConv convolution operation is added. A key feature of AKConv is that the size and shape of its adaptive convolution kernel can be dynamically adjusted according to actual needs [46,54]. This design reduces the original parameters from a quadratic growth to a linear growth, significantly decreasing the computational load; the structure of this module is shown in Figure 5. After the DCNv-C2f layer completes the initial feature representation, AKConv carries out feature fusion. AKConv dynamically generates convolutional kernel weights, adapting the convolution parameters based on the context of the input feature map. This mechanism enhances the model's ability to express multi-scale features and suppresses background noise. The backbone network aims to retain as much crack detail information as possible, with the collaborative enhancement mechanism of DCNv-C2f and AKConv, where the former handles local convolution modeling and the latter is responsible for multi-scale feature fusion. They share parameters and complement each other, jointly improving the model's ability to extract complex geometric features of cracks. Additionally, AKConv utilizes SiLu as the activation function [55,56], enabling the model to learn more complex feature representations, adapt to variations in local features, and handle crack images in occluded and complex background environments.

(3):The Global Attention Mechanism (GAM) operates at the head of the neck network. The final layer of the backbone network, the SPPF module, transforms features of various scales into fixed-size feature vectors. These feature vectors are received by the GAM attention mechanism, which effectively retains the crack information extracted by the backbone network and transmits it to the neck network for subsequent feature fusion, thereby enhancing the neck network's feature fusion capability. When faced with complex background environments (such as occlusions and small target objects), this design significantly improves the model's ability to capture crack features, enhancing its detection performance in complex backgrounds and multi-scale scenarios. This considerably addresses the challenges posed by background environments in road crack detection. Given the complex background with multiple interferences and the irregular geometric characteristics of cracks, the Global Attention Mechanism (GAM) emerges as the optimal choice for suppressing unimportant information in images while extracting significant information.

**[comment]**The paper introduces several advanced techniques (e.g., GAM, AKConv) to enhance detection accuracy. However, it would be helpful to include a brief discussion on the interpretability of the model. How can users or practitioners understand and interpret the decisions made by the model, especially in edge cases where false positives or negatives occur? Providing insight into the model’s decision-making process could enhance its acceptance in real-world applications.

**[Reply]**

Thank you for your valuable comments. By using the gradient weighted class activation mapping (grade CAM) technology to understand the decision-making process of neural network, we visualized the image region in the baseline model and Flexi Yolo that contributed the most to the prediction, significantly improved the transparency and trust of the model, and made the decision-making process of the model easier to explain

**[Modified]**

To enhance the model's interpretability and effectiveness, we conducted a visual analysis of the SPPF layer of yolov8n and the GAM attention mechanism layer of the Flexi-YOLO model using Gradient-weighted Class Activation Mapping (Grad-CAM) [62]. Grad-CAM computes gradients based on class confidence scores and generates corresponding weights. This approach allows for an intuitive understanding of the feature representation among different modules and network layers, particularly regarding the focus of features in the complex task of detecting road cracks in cluttered backgrounds when using Flexi-YOLO.

Fig : Thermodynamic diagram

The experimental results are shown in Fig. 16. The SPPF layer of the backbone network of the baseline model aggregates multi-scale contextual features through spatial pyramid pooling, and its fixed receptive field restricts the adaptability to irregular crack patterns. In contrast, Flexi-YOLO focuses on feature details through its convolution operation with adaptive sampling points and the GAM attention mechanism, enabling the model to focus more on the target region.Specifically, when the original image presents cracks in streaks or grids, the baseline model can only focus on the central parts of the cracks and fails to address the edges. Additionally, when cracks bifurcate, the baseline model only recognizes parts of one crack while neglecting the other bifurcated portion. On the other hand, Flexi-YOLO benefits from its excellent adaptive sampling mechanism, which dynamically adjusts the positions of sampling points based on the shape of the cracks. In contrast, the GAM attention mechanism retains important features and suppresses ineffective ones. By visualizing the model's heat maps, the experimental results further illustrate the reasons for and effectiveness of Flexi-YOLO in avoiding missed detections and false positives.

Finally, the authors would like to express their gratitude to the reviewers for the valuable comments, and the time and efforts they have spent in the review. Without the expert comments made by the reviewers, the paper would not be of this quality.
