# Supplementary material for: Flexi-YOLO: A lightweight method for road crack detection in complex environments
Source: PLoS One. 2025 Jun 16;20(6):e0325993. doi: 10.1371/journal.pone.0325993 (PMC12169580; doi:10.1371/journal.pone.0325993)
Supplement: Attachment — Submitted filename: Response_to_Reviewers_auresp_2.docx [file pone.0325993.s002.docx]

Response to Reviewers

**Title:** **Flexi-YOLO: A Lightweight Method for Road Crack Detection in Complex Environments**

**Manuscript Number: PONE-D-24-58741**

**Author:** **Jiexiang Yang ^*,+^, Renjie Tian ^+^, Zexing Zhou , Xingyue Tan and Pingyang He**

First of all, the authors would like to express their sincere thanks to the reviewers and editors for helpful comments and suggestions. The explanation of the modifications as well as corrections in this revision can be arranged as follows.

The comments of the reviewers are listed in [comment] in italics below. Our reply is in normal font in [reply]. The modified content is given in [modified] in normal font. The modification details are tracked in detail with the latexdiff given on the official website and named Revised Manuscript with Track Changes.pdf . The modifications or additions to the manuscript are marked in blue text.

**Response to Reviewer #1**

We sincerely thank you for your thorough review and valuable suggestions regarding our article. After carefully studying and implementing each of your proposed revisions, we are pleased to find that the quality of the article has significantly improved from its initial version. Your professional comments have helped us avoid numerous shortcomings and research pitfalls, resulting in a more rigorous academic expression and a more coherent structure. We have made every effort to revise based on your feedback and hope that you will recognize our efforts.

Once again, we express our heartfelt gratitude; your insights will have profound implications for our future research endeavors.

**[Comment]**

*Language Correction:
Please see carefully the comments and give reply. All the comments are not answered satisfactorily.Literature review should be strengthened and find the critical gap of literature.*

**[Reply]**

Thank you for your valuable feedback on our manuscript. We deeply recognize the shortcomings in the critical discussion of the literature in our previous revisions, as well as our failure to adequately address the key issues you raised. We sincerely apologize for this. Consequently, we have systematically re-evaluated and revised the literature in response to the inappropriate modifications mentioned in your last review. We have placed special emphasis on your important suggestion to "enhance the literature review and identify critical gaps," and the revised content is presented in the following sections.

**[Comment]**

*Language Correction:*

*Abstract:*

*Rephrase for conciseness and impact. For instance:*

*"Road crack detection is vital for infrastructure maintenance and public safety, yet complex backgrounds and irregular crack shapes challenge real-time, efficient, and accurate detection. This paper proposes Flexi-YOLO, a lightweight and robust model based on YOLOv8, incorporating novel components to address these challenges. Key innovations include the Wise-IoU loss function for enhanced regression accuracy and robustness, the DCNv-C2f module for feature transformation, and the Global Attention Module (GAM) for improved global information perception. Flexi-YOLO achieves significant improvements in accuracy, recall, and mAP metrics, offering a cost-effective solution for automated crack detection."*

**[Reply]**

Thank you very much for your suggestion. We have rephrased the summary to make it more concise and highlight the core impact.

**[Modified]**

Road crack detection is critical to global infrastructure maintenance and public safety, and complex background environments and nonlinear damage crack patterns challenge the need for real-time, efficient, and accurate detection.This paper proposes a lightweight yet robust Flexi-YOLO model based on the YOLOv8 algorithm. We designed Wise-IoU as the model's loss function to optimize the regression accuracy of its bounding boxes and enhance robustness to low-quality samples. The DCNv-C2f module is constructed for the transformation and fusion of feature information, allowing the convolutional kernels to adapt to the complex shape characteristics of cracks dynamically. A Global Attention Module (GAM) is integrated to improve the model's perception of global information. The AKConv convolution operation is employed to adaptively adjust the size of convolutions, further enhancing local feature capturing. Additionally, a lightweight network design is implemented, establishing G-Head (Ghost-Head) as the detection head to optimize the issue of feature redundancy. Experimental results show that Flexi-YOLO achieves an accuracy increase of 2.7% over YOLOv8n, a recall rate rise of 4.7%, a mAP@0.5 improvement of 5.3%, a mAP@0.5-0.95 increase of 3.9%, a decrease of 0.5 in GFLOPS, and an F1 score improvement from 0.80 to 0.84. Flexi-YOLO offers higher detection accuracy and robustness and meets the industrial demands for lightweight real-time detection and lower application costs, providing an efficient and precise solution for the automated detection of road cracks.

**[Comment]**

*Related Work:*

*Ensure smooth transitions between traditional methods and deep learning advancements.*

**[Reply]**

Thank you for your professional comments on our manuscript. We have re-optimized the introduction and related work sections, adding several transitional elements to ensure a smoother and more natural flow between traditional methods and deep learning approaches.

**[Modified]**

**(1) Introduction:**

In the field of crack detection, it is usually divided into two stages. The first stage involves using traditional methods for detection. However, with the advancement of artificial intelligence, methods utilizing computer vision are gradually replacing traditional techniques. Although mainstream deep learning methods in road crack detection have made significant progress, they still face several key challenges. Factors such as background noise, surface texture, lighting shadows, crack edges, and variations in crack shape, size, depth, and moisture often lead to false positives and missed detections, increasing the difficulty of detection [9]. These factors require models to not only adapt to different background noises but also possess the ability to handle surface textures and shadows, enhancing their resistance to interference. Road safety work is often conducted on mobile devices that typically have limited computational power and lower application costs. Therefore, it is necessary to ensure that the models are lightweight and flexible enough to adapt to such devices.

**(2) Related Work:**

①Traditional methods:

Manual inspection and visual examination in crack detection remain the primary methods in most developing countries. However, manual inspection faces a series of issues, including but not limited to low efficiency, subjective judgment factors affecting assessments, difficulty in detecting fine cracks with the naked eye, high labor costs, and significant errors [10]. Thermal imaging technology has also garnered attention for crack detection due to its high portability and insensitivity to lighting conditions, offering specific advantages; however, this method is quite expensive, and its resolution needs improvement [11]. Ultraviolet and laser techniques [10][12]are frequently used in the detection of pavement cracks, as they can produce high-resolution images. However, this approach requires complex equipment and has high costs. Ground Penetrating Radar (GPR) is a non-destructive testing method widely used for pavement inspection [13][14], but it generates large amounts of data, has slow processing times, and suffers from subjective data interpretation and inconsistent standards. While these traditional techniques each have advantages, none have successfully balanced the issues of accuracy, efficiency, and cost.

In recent years, with the rapid development of computer vision, researchers have provided new technological directions for crack detection, which can be broadly categorized into two types. The first type is based on traditional digital image processing, primarily involving manual feature discrimination, where specific feature recognition criteria are designed to facilitate identification[15-17]. Kim J T et al. [18] utilized structural frequency changes to detect and locate cracks, while Nguyen et al. [19] employed the geometric properties of cracks in images to identify their edges. Jin et al. (2018) [20] proposed a method for detecting pavement cracks by integrating directional gradient histograms with the watershed algorithm. However, traditional image processing methods struggle to meet crack detection's accuracy and time requirements [21]. It is noteworthy that while traditional crack detection methods achieved some success compared to manual inspections in earlier stages, their heavy reliance on human intervention and fixed detection scenarios has led to persistent issues such as inefficiency and poor generalization performance, prompting researchers to explore alternative approaches. The second type is based on deep learning theory, utilizing convolutional neural networks for autonomous feature learning, thereby achieving the task of crack detection. Due to its high levels of automation, intelligence, precision, and robustness, this approach has become the mainstream method in the detection field and is widely adopted by scholars[22-24].

②Deep Learning Methods:

Deep learning methods have addressed some issues of traditional crack detection techniques, reducing detection errors caused by human subjectivity and overcoming the limitations of conventional image processing methods in recognizing complex backgrounds in practical engineering applications.

**[Comment]**

*Expand on limitations of existing YOLO-based methods and how Flexi-YOLO surpasses them.*

**[Reply]**

Thank you for your attention and thorough review of our manuscript. In response to the limitations and shortcomings of the YOLO algorithm, we have added a more comprehensive literature review at the end of the related work section to illustrate these limitations. Additionally, we have briefly described the content and innovations of our work to highlight the advantages of Flexi-Yolo.

**[Modified]**

Sapkota et al. [47] found that, when compared to YOLOv8, YOLOv9, and YOLOv10, YOLOv8 maintains accuracy and inference speed while achieving a lower computational cost. However, despite its strong performance in multi-scale feature extraction, YOLOv8 still struggles with detecting small objects and defect cracks, leading to frequent missed detections and false positives. The primary issue is that as the network layers increase, some shallow feature information becomes difficult to retain [48].

One-stage algorithms provide faster detection speeds in real-time scenarios but sacrifice some level of accuracy. The optimization efforts in the studies mentioned above mainly focus on the backbone and neck networks, overlooking the potential of the detection head in model optimization. While these works have achieved remarkable outcomes, there are still deficiencies in balancing the model for detection tasks in non-ideal backgrounds and the pursuit of lightweight solutions. To address the limitations of deep learning methods in lightweight crack detection tasks under complex backgrounds, we aim to enhance the model's feature extraction and fusion capabilities by redesigning the backbone and neck network structures. Additionally, we propose an innovative detection head to fill the gap in optimizing the detection head to achieve model lightweightness, thereby catering to the adaptability of edge computing or mobile infrastructures.

**[Comment]**

*More Literature Review:
Discuss advancements in deep learning for infrastructure health monitoring, such as applications beyond road cracks (e.g., bridge, pipeline, and building inspections).*

**[Reply]**

We believe this is a well-considered and professional opinion. Based on your feedback, we have carefully reviewed the relevant literature and elaborated on the applications of crack detection in other fields within the related work section. By examining crack detection methods from other disciplines, we have made our manuscript more comprehensive and persuasive.

**[Modified]**

Cha et al.[32] used CNN in combination with the sliding window technique to scan the test images to make the model excel at detecting thin cracks under illumination conditions. Hacıefendioğlu et al.[33] used Faster R-CNN to study crack detection to conclude that lighting conditions have the highest impact on crack detection. Xu et al.[34] found that the former is better than the latter under certain conditions by comparing the detection of cracks with Faster R-CNN and Mask R-CNN, but the two algorithms require large and complex datasets. To address these problems, researchers have proposed various optimization strategies. For example, Zhang et al.[35] introduced MobileNetV2 and CBAM into YOLOv3 to reduce the network parameters. Yu et al.[36] pruned YOLOv4 to significantly increase the detection rate while ensuring the accuracy to contribute to the UAV crack detection. Chen et al.[37] introduced the Ghost module and CA attention mechanism in YOLOv5 for the backbone network to effectively improve the detection of coal cracks, which contributes to the construction of intelligent mines. Chen et al.[38] proposed a sample enhancement strategy based on cycle-GAN, which uses a migration learning strategy to incorporate the Transformer attention mechanism in YOLO v5 to help the detection of cracks by drones. Transformer attention mechanism, to help the network find the region of interest in the complex background, to improve the detection and identification of small-scale defects, for the pipeline defects task to solve the overfitting problem brought by small samples and large models. Tran et al.[39] compared the mainstream models, selected YOLOv7, and proposed a U-Net model that can detect and segment bridge deck cracks in a fast and high-precision way. detection and segmentation of bridge surface cracks. Xiong et al.[40] incorporated the GAM attention mechanism and Wise-IoU into YOLOv8 to study the bridge surface cracks and found that the GAM attention mechanism has a great effect on crack detection. Wang et al.[41] optimized YOLOv8 with simSPPF using the spatial pyramid pooling layer in YOLOv8 and introduced the dynamic large convolutional kernel LSK attention mechanism, making the model more effective for pavement defect detection. Liu et al. [42]introduced the C2f_AK module in the neck network to enhance feature fusion, significantly improved the detection head, and reduced parameters to achieve model lightweighting.

**[Comment]***Include recent works utilizing YOLO-based architectures in different domains to position Flexi-YOLO in the broader context.*

**[Reply]**

Thank you very much for your suggestions; they have been immensely helpful for our manuscript. Your insights have broadened our perspective on the applications of YOLO technology across various fields, allowing our article to extend beyond just crack detection. Following the style of the reference papers you provided, we have included more recent literature in the related work section to further explore the applications of YOLO in different domains.

**[Modified]**

It is noteworthy that YOLO has not only achieved results in the field of crack detection but also in agricultural applications. Di et al. [43] proposed the DF-Tiny-YOLO model for apple leaf disease detection, effectively addressing the issue of distinguishing between healthy and diseased apple leaves. Liu et al. [44] introduced the ETSR-YOLO model to tackle the challenge of traffic sign recognition in road environments, providing a new solution for traffic sign recognition on embedded platforms in vehicles. Gong et al. [45] developed a two-stage ship detection method based on YOLOv8 for long-distance vessel detection, offering new insights into the field. Xu et al. [46] designed three plug-and-play modules—DGLFG, SCCFF, and ISPP—based on YOLOv8, significantly improving the feature learning capabilities for detecting small objects from aerial photography.

**[Comment]**

*Highlight shortcomings of current methods, like high computational requirements, sensitivity to noise, or lack of adaptability to varying crack shapes.*

**[Reply]**

Thank you for your suggestion. Based on the modifications we made to the original YOLOv8 Algorithm's conclusion, we have added other deep learning methods and the limitations of YOLO in the related work section.

**[Modified]**

**(1) Related work:**

①Deep learning-based detection can be broadly classified into two categories: two-stage detection algorithms and single-stage detection algorithms. The former first generates a series of candidate regions and then classifies these regions and performs bounding box regression. Representative models include R-CNN, Fast R-CNN, Faster R-CNN, and Mask R-CNN [25-28]. The latter directly predicts classes and bounding boxes on the image without the need to generate candidate regions, with models such as You Only Look Once (YOLO) and SSD being representative [29-30]. However, existing deep learning methods still face numerous challenges, as most models have high computational complexity and slow processing speeds, which limit their application scenarios [31]). Many models are sensitive to environmental noise and prone to false positives in noisy backgrounds, and there is limited exploration of application scenarios across different domains. To address these issues, researchers have proposed various optimization strategies.

②Sapkota et al. [47] found that, when compared to YOLOv8, YOLOv9, and YOLOv10, YOLOv8 maintains accuracy and inference speed while achieving a lower computational cost. However, despite its strong performance in multi-scale feature extraction, YOLOv8 still struggles with detecting small objects and defect cracks, leading to frequent missed detections and false positives. The primary issue is that as the network layers increase, some shallow feature information becomes difficult to retain [48].

**(2)YOLOv8 Algorithm:**

Despite the exceptional performance of YOLOv8, effectively handling occluded objects and cluttered scenes and adapting to overlapping or various crack shapes remains a long-term challenge for the YOLO family [50].

**[Comment]**

*Incorporate examples of lightweight models applied in mobile and edge computing for infrastructure maintenance, emphasizing how Flexi-YOLO aligns with these trends.*

**[Reply]**

Thank you for noticing this detail and providing valuable guidance. We have introduced a more extensive literature review from related fields in the relevant work section, exploring lightweight achievements that lead into how Flexi-YOLO achieves model optimization by enhancing the detection head for lightweight applications. We also examined the parameters of the YOLOv8 model, with experimental results demonstrating that the decoupled detection head design represents a significant portion of the model's parameters, indicating considerable optimization potential for the detection head. Consequently, we are focused on redesigning the detection head to reduce the model's weight. The new detection head significantly minimizes the information redundancy caused by the attention mechanism's excessive collection of global information and reduces the parameter count of the original detection head, making the model more lightweight and suitable for low-computation environments. At the end of the related work section, we emphasize how Flexi-YOLO should align with these trends.

**[Modified]**

One-stage algorithms provide faster detection speeds in real-time scenarios but sacrifice some level of accuracy. The optimization efforts in the studies mentioned above mainly focus on the backbone and neck networks, overlooking the potential of the detection head in model optimization. While these works have achieved remarkable outcomes, there are still deficiencies in balancing the model for detection tasks in non-ideal backgrounds and the pursuit of lightweight solutions. To address the limitations of deep learning methods in lightweight crack detection tasks under complex backgrounds, we aim to enhance the model's feature extraction and fusion capabilities by redesigning the backbone and neck network structures. Additionally, we propose an innovative detection head to fill the gap in optimizing the detection head to achieve model lightweightness, thereby catering to the adaptability of edge computing or mobile infrastructures.

**[Comment]***Literature references suggested for manuscript optimization,*

*You can follow：
[1] S. Mandal, A. Shiuly, D. Sau, A. K. Mondal, and K. Sarkar, “Study on the use of different machine learning techniques for prediction of concrete properties from their mixture proportions with their deterministic and robust optimisation,” AI Civ. Eng., vol. 3, no. 1, p. 7, Dec. 2024, doi: 10.1007/s43503-024-00024-8.
[2] K. Sarkar, A. Shiuly, and K. G. Dhal, “Revolutionizing concrete analysis: An in-depth survey of AI-powered insights with image-centric approaches on comprehensive quality control, advanced crack detection and concrete property exploration,” Constr. Build. Mater., vol. 411, p. 134212, Jan. 2024, doi: 10.1016/j.conbuildmat.2023.134212.
[3] A. Shiuly, D. Dutta, and A. Mondal, “Assessing compressive strengths of mortar and concrete from digital images by machine learning techniques,” Front. Struct. Civ. Eng., vol. 16, no. 3, pp. 347–358, Mar. 2022, doi: 10.1007/s11709-022-0819-z.*

**[Reply]**

We sincerely appreciate your valuable feedback. We have thoroughly reviewed the references and added citations for these three papers in the introduction and related work sections of the revised manuscript. First, in the introduction, we transition to applications in other fields by referencing [2]. We explore that research on infrastructure security can be approached from two directions: on one hand, by enhancing the inherent properties of materials to improve structural durability; on the other hand, through timely crack detection to prevent disasters. In this discussion, we highlight the importance of the first method by referencing [1] and [3], which leads us to the second approach—computer vision-based non-destructive testing methods. In the related work section, we examine some shortcomings of current deep learning methods and use [3] to demonstrate innovative cases of deep learning in other underexplored areas, thereby addressing the previous comments about the lack of exploration in diverse field applications.

**[Modified]**

**(1) Introduction:**

Roads are a critical component of global infrastructure and are essential for the long-term stable development of the world economy. However, due to prolonged use and various environmental factors, infrastructure structures inevitably experience aging, with concrete structures particularly susceptible to cracking [1]. These cracks affect road durability, safety, and usability, reduce their load-bearing capacity, shorten their lifespan, and even threaten user safety [2].Structural Health Monitoring (SHM) has expanded into diverse fields, including bridges, pipelines, tunnels, and buildings. However, limitations remain in Structural Damage Identification (SDI) and precise crack detection and analysis. Sarkar et al. [3] address this gap by exploring the development and application of Artificial Intelligence in civil engineering, focusing on concrete structures. Their research on concrete provides methods and conclusions applicable not only to road crack detection but also to the health monitoring of other infrastructure. According to the World Health Organization's 2023 report, approximately 1.19 million people die each year due to road traffic accidents, with nearly 3,400 deaths per day and up to 50 million injured. Among these, individuals aged 5 to 29 represent the primary age group for road accident fatalities, which is also a crucial demographic for nations' current and future economic development, bearing the responsibility for significant economic construction.

If road cracks are not maintained or repaired, minor cracks can develop into deeper fissures and potholes, leading to greater economic losses. As one of the primary materials used in infrastructure such as roads, bridges, and tunnels, the structural health of concrete is a significant concern for these facilities. Enhancing concrete's inherent strength and durability to improve the safety of foundational structures is a reliable approach [4]. Mandal et al. [5] utilized machine learning techniques to predict the performance of concrete based on the mixing ratios of its components, while Shiuly et al. [6] applied various machine learning methods to forecast the compressive strength of mortar and concrete. Their research provides cost-effective preventive measures for infrastructure safety from the material perspective. Crack detection is a key indicator for assessing the condition of concrete structures and monitoring the safety and maintainability of infrastructure. Employing computer vision for non-destructive testing (NDT) of cracks is another mainstream method [3], which plays a crucial role in subsequent road maintenance and repairs. Therefore, timely detection and assessment of road cracks are necessary to reduce later repair and maintenance costs [7].

**(2) Related Work:**

Deep learning-based detection can be broadly classified into two categories: two-stage detection algorithms and single-stage detection algorithms. The former first generates a series of candidate regions and then classifies these regions and performs bounding box regression. Representative models include R-CNN, Fast R-CNN, Faster R-CNN, and Mask R-CNN [25-28]. The latter directly predicts classes and bounding boxes on the image without the need to generate candidate regions, with models such as You Only Look Once (YOLO) and SSD being representative [29-30]. However, existing deep learning methods still face numerous challenges, as most models have high computational complexity and slow processing speeds, which limit their application scenarios [31]. Many models are sensitive to environmental noise and prone to false positives in noisy backgrounds, and there is limited exploration of application scenarios across different domains. To address these issues, researchers have proposed various optimization strategies. Shiuly et al.[6]compared the effectiveness of six models in predicting the compressive strength of concrete, demonstrating the efficacy of deep learning models in predicting material performance and providing new insights for the application of deep learning in structural performance assessment.

**[Comment]**

*Language Refinement:
Avoid repetitive phrases like "complex backgrounds" and "irregular crack shapes." Use synonyms or restructure sentences for variety.
Use active voice for clarity and engagement. For example:
"Flexi-YOLO addresses the limitations of previous methods by leveraging advanced convolutional techniques and attention mechanisms."*

**[Reply]**

Thank you very much for your suggestions. We carefully re-evaluated the article, reorganized it, and replaced excessively repetitive phrases such as "complex backgrounds" and "irregular crack shapes" with alternatives like "highly disturbed environments," "multi-interference backgrounds," "chaotic backgrounds," "interference scenes," "geometric variability," "geometric features," and "nonlinear expansion characteristics" to enhance the readability of the article. We also meticulously reviewed the adherence to language norms and made improvements under strict regulatory standards. Additionally, we employed the active voice more frequently when articulating the study's innovations and conclusions to emphasize clarity and engagement in our writing. This optimization has made the article more aligned with the writing conventions of native English speakers. We greatly appreciate the reviewers’ precise observations and suggestions regarding details, which have contributed to the refinement of our article.

**[Comment]**

*Conclusion:
Reiterate the novelty and potential applications of Flexi-YOLO, emphasizing its real-world impact. Example:
"Flexi-YOLO represents a significant step forward in automated road crack detection. Its lightweight design, coupled with state-of-the-art feature extraction and attention mechanisms, ensures high accuracy and adaptability in diverse environments. This model not only meets the demands of real-time applications but also offers a cost-effective solution for infrastructure maintenance, contributing to public safety and sustainability."*

**[Reply]**

Thank you for your thorough review of our manuscript and for your valuable comments that will help us optimize it. In the conclusion section, we reiterate the significance of this study for global infrastructure maintenance and the superiority of our model. We highlight the novelty of Flexi-YOLO and its potential applications, which not only represent a technological advancement in the field of global road defects research but can also be extended to infrastructure maintenance in other sectors.

**[Modified]**

This article presents a novel Flexi-YOLO model applied to road crack detection. The model employs DCNv-C2f adaptive convolution sampling positions and AKConv adaptive convolution kernel sizes within its backbone network to enhance its ability to extract crack features, allowing for more accurate and flexible crack identification. By adding a GAM attention mechanism at the neck of the network, the model can comprehensively capture global information and further fuse detailed features with low-level network characteristics to obtain more feature information. The newly designed detection head mitigates the issue of information redundancy caused by the attention mechanism's extensive global information collection and the increased model size due to the high parameter count and computation in the original detection head, making it challenging to deploy on devices with limited computational resources. The Wise-IoU method assigns different weights based on sample quality, effectively reducing the negative impact of low-quality samples on the model. With these improvements, the YOLOv8 model performs better in handling complex information across various road conditions, demonstrating stability against fine details and interference information. The developed model shows outstanding performance across various metrics, specifically a 2.7% increase in accuracy, a 4.7% increase in recall, a 5.3% improvement in mAP@0.5, a 3.9 improvement in mAP@0.5-0.95, a 0.5 decrease in GFLOPS, and an increase in F1 score from 0.80 to 0.84. This data underscores the superiority of Flexi-YOLO in multiple dimensions. Furthermore, practical detection experiments indicate that our model excels in numerical evaluation and real-world road condition detection. This validates the practicality of our model improvements and provides valuable experience and technological advancement for real-world road crack operations, offering reliable solutions for global road aging, safety, and maintenance. Its lightweight design also presents an economically efficient solution for infrastructure maintenance, contributing to global public safety and sustainable development.

**Response to Reviewer 2**

**[Reply]**

Thank you very much for your affirmation and recognition of our research work. Your acknowledgment serves as a tremendous encouragement on our scientific journey. Your constructive feedback has helped us refine our research content and enhance the quality of our paper. With a deep respect for academia, we will continue to strive for exploration in our research endeavors. Once again, we sincerely appreciate your valuable time and professional guidance.

**Response to Reviewer 3**

Dear Reviewer, I sincerely thank you for recognizing our work. Your acknowledgment is a tremendous encouragement for our research efforts. Your professional suggestions have greatly contributed to the quality of this article. Once again, thank you for your constructive feedback. Wishing you all the best.

**[Comment]**

*Please provide clear links and/or references to the source dataset Roboflow crack dataset for full credibility and reproducibility.*

**[Reply]**

Thank you for your careful review of our manuscript, and we sincerely apologize for the oversight regarding this detail. In the Data set section, we have added a reference link for the Roboflow crack dataset. As for the Data6000 dataset in the Model Generalization Experiment section, it is composed of multiple datasets integrated from Roboflow, making it difficult to trace back to specific links. We apologize for this as well; however, we have uploaded the Data6000 dataset and all datasets mentioned in this paper to a public database for academic scrutiny and rigorous research. The specific URL is <https://doi.org/10.5281/zenodo.15019275>.

**[Comment]**

*The section of “Model Generalization Experiment”, on page 24, is considerate and well-executed, however, adding additional details about the class distribution within the datasets would be helpful.*

**[Reply]**

Thank you for your recognition of our work and for the highly valuable feedback you provided. We have re-evaluated the classification within the dataset to ensure the rigor of our article's narrative, and we have presented the findings in a table in the Model Generalization Experiment section.

**[Modified]**

| Dataset | Category | Annotation Count |
| --- | --- | --- |
| Data6000 | Crack | 9120 |
| MotorBrike | D00 | 2678 |
|  | D10 | 1096 |
| Drone | D00 | 1421 |
|  | D10 | 1263 |

Table 5: Distribution of Dataset Annotations

The Data6000 dataset consists of 6,000 images, while the MotorBike dataset contains 1,934 images, and the Drone dataset has 1,917 images. The internal annotation distribution of these different datasets is shown in Table 5. The annotation quantities for the D00 and D10 categories are relatively balanced, which aids the model's comprehensive feature learning, enhancing its generalization capability and accuracy in recognition. This helps effectively mitigate various negative impacts caused by insufficient data. A sufficient amount of labeled data provides a reliable foundation for feature extraction and parameter optimization during the model training process, enabling Flexi-YOLO to maintain stable detection performance during evaluations.

**[Comment]***The section of “Computational Analysis of Low-Power Devices”, on page 26 and 27, mentions good performance on hardware test. Could you expand this section by including quantitative metrics such as power consumption, latency during inference, memory consumption, and frames per second? This guarantees the sustainability and suitability for edge deployment.*

**[Reply]**

Thank you very much for your thorough review of our article. Your suggestion regarding the quantitative analysis of the model is very constructive. Due to our oversight, we neglected to include this crucial piece of information, and we sincerely appreciate your reminder. To more intuitively showcase the excellent performance of Flexi-YOLO, we have added some quantitative metrics in the section titled "Computational Analysis of Low-Power Devices." However, for certain metrics, such as power consumption, we believe the presentation is of limited significance. This is because power consumption is highly dependent on the design characteristics of specific hardware platforms. The same model may exhibit significant differences in power performance when run on different devices, even if the inference performance is similar. This hardware dependency could lead to a lack of generalizability and comparability in power consumption data. In contrast, we consider memory usage and FPS to be more objective performance metrics with less design-related variability across devices, as they can more accurately reflect the practical deployment potential of the model in edge computing environments.

Once again, thank you for your valuable suggestions; they have indeed been constructive in helping us refine the experimental evaluation section of our paper. **[Modified]**

The experimental results shown in Figure 17 indicate that the model exhibits outstanding inference capabilities on the RTX 1650 graphics card. In practical detection scenarios, the model demonstrates good robustness and detection accuracy. The baseline model, however, exhibited many false positives and missed detections in real-world environments, and its performance in locating cracks and forming detection anchor boxes was less than satisfactory. In contrast, Flexi-YOLO performed exceptionally well in practical detection environments. It could accurately identify the position and shape of road cracks even under challenging conditions, such as intense light, shadows, and mottled backgrounds. It successfully categorized multiple cracks in road images into distinct subsets, with a lower occurrence rate of false detections. Furthermore, its detection accuracy surpassed that of the baseline model. Regarding resource consumption, Flexi-YOLO occupies only 1135 MiB of memory, well below the entry-level memory of various mainstream mobile devices (4GB), achieving a real-time processing speed of 59 FPS, significantly exceeding the 30 FPS standard for real-time processing.
